# Monogenic forms of childhood obesity due to mutations in the leptin gene

**DOI:** 10.1186/s40348-014-0003-1

**Published:** 2014-09-04

**Authors:** Jan-Bernd Funcke, Julia von Schnurbein, Belinda Lennerz, Georgia Lahr, Klaus-Michael Debatin, Pamela Fischer-Posovszky, Martin Wabitsch

**Affiliations:** Division of Pediatric Endocrinology and Diabetes, Department of Pediatrics and Adolescent Medicine, Ulm University Medical Center, Ulm, 89075 Germany; Department of Pediatrics and Adolescent Medicine, Ulm University Medical Center, Ulm, 89075 Germany

**Keywords:** Leptin deficiency, Leptin mutation, Leptin secretion, Obesity, Bioinactive hormone

## Abstract

Congenital leptin deficiency is a rare autosomal recessive monogenic obesity syndrome caused by mutations in the leptin gene. This review describes the molecular and cellular characteristics of the eight distinct mutations found so far in humans.

## Introduction

During the last four decades, the prevalence of obesity in childhood has increased dramatically and obesity has become an epidemic disease with more than 5% of all children affected in developed countries [1]. Changes in living conditions may act on children's behavior and favor the development of obesity due to individual genetic susceptibility [1].

In the vast majority of obese children, no syndromal or monogenic cause for the obese state can be diagnosed and therefore a polygenic cause is suggested. Generally, monogenic forms of childhood obesity are very rare [2]. Mutations in only a few genes are known to cause the development of severe obesity in early childhood [2]. Most of these genes are involved in the central nervous regulation of hunger and satiety where the leptin/leptin receptor system plays a pivotal role [3]. Of all monogenic forms of obesity, the only one causally treatable is congenital leptin deficiency caused by homozygous mutations of the leptin gene [4]. Leptin is a protein secreted mainly by adipocytes, and its circulating levels correlate positively with the body mass index and the body fat mass. By central as well as peripheral action, leptin impacts diverse physiological processes including energy balance, metabolism, endocrine regulation, and immune function [3]. One of the main functions of leptin is to control the body fat mass by inhibiting food intake via the central nervous system. Recombinant human leptin (metreleptin) can be administered to patients with congenital leptin deficiency to compensate the lack of leptin [5].

Current clinical recommendations suggest that children with a normal birth weight but rapid weight gain in the first few months of life leading to extreme obesity should be tested for congenital leptin deficiency when they show impaired satiety, intense hyperphagia, and food-seeking behavior (see Clinical phenotype of patients with congenital leptin deficiency) [6,7]. Most patients described up to now had consanguine parents. Patients usually develop metabolic and hormonal alterations including hyperinsulinemia, insulin resistance, severe liver steatosis, dyslipidemia, as well as hypogonadotropic hypogonadism (see Clinical phenotype of patients with congenital leptin deficiency) [7,8]. Furthermore, some patients display immunological alterations in early childhood that manifest in recurrent severe bacterial infections possibly resulting in death in childhood (see Clinical phenotype of patients with congenital leptin deficiency) [4]. Upon substitution therapy with recombinant human leptin applied by daily subcutaneous injections, patients lose weight, the body fat mass is reduced, and the metabolic, hormonal, and immunological abnormalities are normalized [4,8,9].

### Clinical phenotype of patients with congenital leptin deficiency

- Normal birth weight
- Rapid weight gain after birth
- Severe early-onset obesity
- Impaired satiety
- Intensive hyperphagia
- Constant food-seeking behavior
- Recurrent severe and possibly lethal bacterial infections in early childhood (some patients)
- Development of hyperinsulinemia
- Development of severe liver steatosis
- Development of dyslipidemia
- Hypogonadotropic hypogonadism

In humans, eight distinct leptin mutations have been described in the literature, which lead to congenital leptin deficiency when present in the homozygous state. On the cellular level, these mutations result in defects in the synthesis and/or secretion of leptin. In the following paragraphs, we have summarized the molecular and cellular characteristics of these mutations.

## Review

### Leptin gene and protein structure

The gene encoding leptin (*LEP* or *OB*) was discovered by positional cloning in 1994 [10]. The *LEP* gene is located on chromosome 7q31.3 and consists of three exons separated by two introns [11] [Ensembl:ENSG00000174697].

Leptin is a type I cytokine and member of the family of long-chain helical cytokines which also includes interleukin 6 (IL-6), granulocyte colony-stimulating factor (G-CSF), and growth hormone (GH) [12]. It is synthesized as an immature 167-amino acid protein encompassing an N-terminal 21-amino acid signal peptide [10] [Ensembl:ENST00000308868]. Cleavage of the signal peptide yields a non-glycosylated, mature 146-amino acid protein [10] [Ensembl:ENST00000308868]. Its structure features four major α helices A to D as well as a distorted minor α helix E, localized in the loop connecting helices C and D [12]. Assuming an up-up-down-down orientation, the helices A to D form a four-helix bundle which is stabilized by a single intramolecular disulfide bond spanning from the beginning of the loop between helices C and D to the C-terminus of the protein [12].

### Molecular and cellular characteristics of human leptin mutants

**Figure 1:**
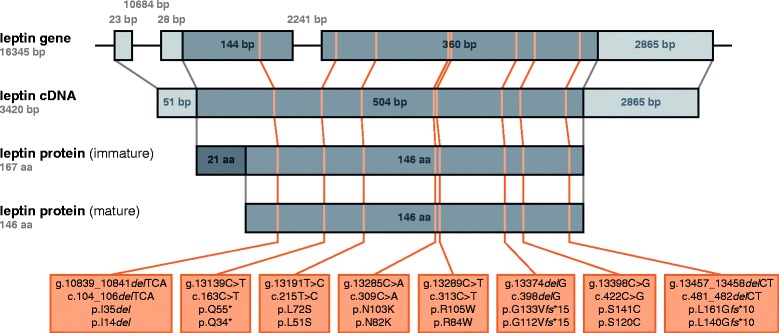
**Overview of human leptin mutants.** Mapping of the eight distinct human leptin mutations to the human leptin gene, leptin cDNA, and immature as well as mature leptin protein. Where applicable, the sizes of individual portions are given either in base pairs (bp) or amino acids (aa). For the leptin gene, exons are depicted as filled boxes while introns and flanking regions are depicted as thin lines. The portions of exon 2 and exon 3 that form the open reading frame (ORF) are colored in a darker shade. For the leptin cDNA, the 5′ and the 3′ untranslated region (UTR) are colored in a lighter shade while the ORF is colored in a darker shade. For the immature leptin protein, the signal peptide that gets cleaved off during the maturation process is colored in a darker shade. For the mature leptin protein, the chosen nomenclature is not consistent with the HGVS recommendations. All sequence information is based on [Ensembl:ENSG00000174697, Ensembl:ENST00000308868].

The notation used to describe the position and character of human leptin mutations has been inconsistent up to now [13-23]. Therefore, we provide a consistent notation and overview of the eight distinct leptin mutations described in humans so far (Figure 1) mapped to the human leptin gene, transcribed leptin mRNA (here cDNA), and translated immature and processed mature leptin protein. In the text of this review, the given position of amino acids in the leptin protein uniformly refers to their position in the unprocessed, immature protein. Moreover, we have summarized information about the eight known leptin mutations in humans, including the number of patients reported to carry these mutations (Table 1). Unless otherwise indicated, the chosen nomenclature adheres to the recommendations of the Human Genome Variation Society (HGVS).

**Table 1:** **Summary of patients reported to carry leptin mutations**

**Mutation**	**Reported patients**	**Serum leptin level**	**Reference**
g.13374*del*G c.398*del*G p.G133Vfs*15 p.G112Vfs*15	30		
	Female, 8 years	0.1 to 1.0 ng/ml (close to detection limit)	Montague et al. [13]
	Male, 2 years	0.4 to 0.7 ng/ml (close to detection limit)	
	Male, 3 years	Below detection limit	Farooqi et al. [5]
	Female, 5 years	Below detection limit	Gibson et al. [24]
	Gender and age not specified	Not reported	Farooqi [25]
	Gender and age not specified	Not reported	
	Gender and age not specified	Not reported	
	Female, 5 months	Below detection limit	Fatima et al. [21]
	Female, 2 years	0.1 ng/ml	
	Female, 2 years	Below detection limit	
	Female, age not specified	Below detection limit	
	Female, age not specified	Below detection limit	
	Male, 1 year	0.5 ng/ml	
	Male, 7 years	Below detection limit	
	Male, 7 years	Below detection limit	
	Male, 12 years	Below detection limit	
	Female, 8 months	0.8 ng/ml	Saeed et al. [22]
	Female, 8 months	1.0 ng/ml	
	Female, 8 months	Below detection limit	
	Female, 2 years	Below detection limit	
	Male, 8 months	Below detection limit	
	Male, 1 year	0.5 ng/ml	
	Male, 1 year 5 months	0.7 ng/ml	
	Male, 3 years 4 months	0.7 ng/ml	
	Male, 7 years	0.9 ng/ml	
	Female, age not specified	Below detection limit	Saeed et al. [26]
	Female, age not specified	Below detection limit	
	Female, age not specified	Below detection limit	
	Female, age not specified	Below detection limit	
	Male, age not specified	Below detection limit	
g.13289C>T c.313C>T p.R105W p.R84W	5		
	Female, 6 years	1.1 ng/ml	Strobel et al. [15]
	Female, 34 years	1.3 to 1.6 ng/ml	
	Male, 22 years	0.9 to 1.2 ng/ml	
	Female, 30 years	0.6 ng/ml	Ozata et al. [27]
	Male, 5 years 1 month	Not reported	Paz-Filho et al. [28]
g.13398C>G c.422C>G p.S141C p.S120C	2		
	Gender and age not specified	Not reported	Chekhranova et al. [17]
	Gender and age not specified	Not reported	
g.13285C>A c.309C>A p.N103K p.N82K	2		
	Female, 7 years	1.3 ng/ml	Mazen et al. [18]
	Male, 3 years	1.1 ng/ml	
g.13191T>C c.215T>C p.L72S p.L51S	1		
	Female, 14 years	0.4 ng/ml (at detection limit)	Fischer-Posovszky et al. [20]
g.10839_10841*del*TCA c.104_106*del*TCA p.I35*del* p.I14*del*	1 to 2 (probable overlap)		
	Female, 7 months	3.6 ng/ml	Fatima et al. [21]
	Female, 1 year 6 months	Below detection limit	Saeed et al. [22]
g.13457_13458*del*CT c.481_482*del*CT p.L161Gfs*10 p.L140Gfs*10	1		
	Male, 1 year 6 months	0.2 ng/ml	Fatima et al. [21]
g.13139C>T c.163C>T p.Q55* p.Q34*	1		
	Female, 8 years	<0.6 ng/ml	Thakur et al. [23]

For the mature leptin protein, the chosen nomenclature is not consistent with the HGVS recommendations. All sequence information is based on [Ensembl:ENSG00000174697, Ensembl:ENST00000308868].

The first mutation found to result in congenital leptin deficiency in humans was originally described in 1997 in two children from a consanguineous Pakistani pedigree [13]. A deletion of the guanine at position 13374 of the leptin gene (g.13374*del*G) corresponding to position 398 of the transcript (c.398*del*G) led to a frameshift mutation of the protein. This changed the glycine at position 133 to a valine and inserted additional 13 subsequent aberrant amino acids before coming to a stop (p.G133Vfs*15) [13]. In both patients, the serum leptin levels as measured by a radioimmunoassay (RIA) and enzyme-linked immunosorbent assay (ELISA) were close to the detection limit (1.0 and 0.7 ng/ml by RIA as well as 0.1 and 0.4 ng/ml by ELISA, respectively) [13]. Furthermore, in one of the patients, no leptin could be detected in the serum by Western blot [13]. Transfecting CHO cells with a plasmid encoding p.G133Vfs*15 leptin showed that p.G133Vfs*15 leptin is synthesized, but not secreted to the cell culture supernatant [13]. Montague et al. proposed two mechanisms responsible for the low serum leptin levels observed in the patients [13]. First, the introduction of a premature stop codon into the transcript might cause nonsense-mediated mRNA decay (NMD) [13]. Second, the introduction of an aberrant stretch of amino acids into the protein might cause improper intracellular transport and secretion [13]. A subsequent detailed study using CHO and COS-7 cells revealed that p.G133Vfs*15 leptin indeed exhibits an improper intracellular transport, aggregates and accumulates in the ER, and subsequently undergoes degradation by the proteasome [14].

In 1998, a second mutation conferring a congenital deficiency in leptin was described in three individuals from a consanguineous Turkish pedigree [15]. A transition of the cytosine at position 13289 of the leptin gene to a thymine (g.13289C>T) corresponding to position 313 of the transcript (c.313C>T) led to a missense mutation of the protein, changing the arginine at position 105 to a tryptophan (p.R105W) [15]. The serum leptin levels as assessed by two different immunoassays were very low in all three patients (1.6, 0.9, and 1.1 ng/ml by the first assay as well as 1.3, 1.2, and 1.1 ng/ml by the second assay, respectively) [15]. Transfecting COS-1 cells, Strobel et al. showed that like p.G133Vfs*15 leptin, p.R105W leptin is synthesized but not secreted [15]. They consequently assumed that the mutant leptin displays a normal synthesis and stability, but aberrant intracellular transport and impaired secretion [15]. Two subsequent studies employing *in vitro*-differentiated PAZ6 adipocytes, primary rat adipocytes, as well as CHO and COS-1 cells confirmed that p.R105W leptin indeed misfolds, aggregates, and accumulates intracellularly [14,16]. In wild-type leptin, p.R105 is surrounded by negatively charged amino acids and forms salt bridges with p.D76 and p.E102 [16]. Boute et al. proposed that the p.R105W mutation might induce protein misfolding by disrupting the formation of these salt bridges [16]. Transfecting COS-1 cells and *in vitro*-differentiated PAZ6 adipocytes with plasmids encoding p.C117S, p.C167S, or p.C117S/C167S leptin, these authors were furthermore able to demonstrate that the correct formation of the intramolecular disulfide bond is a prerequisite for the proper folding, intracellular transport, and secretion of leptin [16]. Boute et al. consequently proposed that the p.R105W mutation might alternatively induce protein misfolding by sterically interfering with the proper formation of this intramolecular disulfide bond [16].

A third mutation was described in 2008 in two individuals from a population inhabiting a small Turkmenian mountain village [17]. A transversion of the cytosine at position 13398 of the leptin gene to a guanine (g.13398C>G) corresponding to position 422 of the transcript (c.422C>G) led to a missense mutation of the protein, changing the serine at position 141 to a cysteine (p.S141C) [17]. No measurement of serum leptin levels was reported in these patients [17]. Chekhranova et al. proposed that the p.S141C mutation might result in protein misfolding and/or loss of biological activity by introducing a third cysteine into the protein, interfering with the correct formation of the intramolecular disulfide bond [17]. To our best knowledge, no study investigating the synthesis or secretion of this mutant has been published yet.

In 2009, a fourth mutation was described in two children from a consanguineous Egyptian pedigree [18]. Here, a transversion of the cytosine at position 13285 of the leptin gene to an adenine (g.13285C>A) corresponding to position 309 of the transcript (c.309C>A) led to a missense mutation of the protein, changing the asparagine at position 103 to a lysine (p.N103K) [18]. The serum leptin levels were very low in both patients (1.1 and 1.3 ng/ml) [18]. Mazen et al. did not explicitly hypothesize about how the p.N103K mutation might impact the synthesis, secretion, or biological activity of the protein [18]. Also in this case, no study examining the synthesis or secretion of this mutant has been published up to now. Notably though, the biological activity of this mutant was nonetheless addressed by an *in vitro* study performed by Niv-Spector et al. [19]. They made use of a prokaryotic expression system to produce p.N103K leptin and demonstrated that the p.N103K mutation drastically reduces the biological activity of the protein [19]. While they do not provide insight into the mechanisms underlying the defective synthesis and/or secretion of the mutant protein, they claim that not only the very serum levels but also the biological inactivity of the p.N103K leptin may contribute to the phenotype observed in affected patients [19].

A fifth mutation was described by our group in 2010 in a child from an Austrian pedigree without known consanguinity [20]. A transition of the thymine at position 13191 of the leptin gene to cytosine (g.13191T>C) corresponding to position 215 of the transcript (c.215T>C) led to a missense mutation of the protein, changing the leucine at position 72 to a serine (p.L72S) [20]. In this patient, the serum leptin levels as measured by an RIA were at the detection limit (0.4 ng/ml) [20]. Moreover, no leptin could be detected in the serum of this patient by immunoprecipitation and subsequent Western blot [20]. Analyzing patient-derived adipose tissue, expression of the mutant protein could be detected in adipocytes by qRT-PCR, Western blot, as well as immunohistochemistry [20]. Furthermore, *in vitro*-differentiated patient-derived adipocytes showed expression of the mutant protein [20]. Transfecting HEK293 cells confirmed that p.L72S leptin indeed is synthesized, but not secreted [20]. By generating plasmids encoding p.L72A, p.L72I, p.L72V, or p.L72T leptin and transfecting these plasmids into HEK293 cells, our group was able to determine the lack of side chain hydrophobicity at position 72 of the protein as the main cause for the lack of secretion [20].

A sixth and a seventh mutation were originally described in 2011 in individuals stemming from consanguineous Pakistani pedigrees [21]. In a child, a deletion of a thymine-cytosine-adenine triplet at positions 10839 to 10841 of the leptin gene (g.10839_10841*del*TCA) corresponding to positions 104 to 106 of the transcript, respectively, (c.104_106*del*TCA) was detected [21]. This in turn resulted in the deletion of the isoleucine at position 35 of the protein (p.I35*del*) [21]. In this patient, Fatima et al. originally observed low, but still detectable serum leptin levels as measured by an ELISA (3.6 ng/ml) [21]. In a later publication, Saeed et al. also reported a child with a p.I35*del* mutation, probably the very same patient, in which they could not detect any leptin in the serum using an ELISA [22]. Fatima et al. hypothesized that the deletion of this highly conserved isoleucine at position 35 of the protein and thus resulting loss of hydrophobicity in the N-terminal region of the protein might result in defective intracellular transport and secretion [21]. Furthermore, these authors proposed that the p.I35*del* mutation might also result in a loss of the biological activity of the secreted protein as the N-terminal region of leptin has been proposed to be involved in the binding of leptin to the leptin receptor [21].

In another child, a deletion of a cytosine-thymine doublet at positions 13457 and 13458 of the leptin gene (g.13457_13458*del*CT) corresponding to positions 481 and 482 of the transcript (c.481_482delCT) led to a frameshift mutation of the protein, changing the leucine at position 161 of the protein to a glycine and inserting additional eight subsequent aberrant amino acids before coming to a stop (p.L161Gfs*10) [21]. In this patient, the serum leptin level as measured by an ELISA was very low (0.2 ng/ml) [21]. Fatima et al. proposed that in the case of the p.L161Gfs*10 mutation, the loss of the C-terminal p.C167 and thus resulting loss of the intramolecular disulfide bond as well as the introduction of an aberrant stretch of amino acids might result in misfolding, impaired intracellular transport and secretion, as well as a loss of the biological activity of the secreted protein [21]. No study examining the synthesis or secretion of either the p.I35*del* or the p.L161Gfs*10 mutant has been published up to now.

Only recently, in 2013, an eighth mutation was described in a child from a consanguineous Indian pedigree [23]. Here, a transition of the cytosine at position 13139 of the leptin gene to thymine (g.13139C>T) corresponding to position 163 of the transcript (c.163C>T) led to a nonsense mutation of the protein, converting the glutamine at position 55 to a stop (p.Q55*) [23]. The serum leptin levels in this patient were very low (<0.6 ng/ml) [23]. Thakur et al. did not explicitly hypothesize about how the introduction of an early premature stop codon into the mutant transcript might impact the synthesis, secretion, or biological activity of the protein [23]. As with the p.G133Vfs*15 and p.L161Gfs*10 mutants, NMD should definitely be considered as a possible mechanism causative of the very low serum leptin levels observed with this mutant. Again, to our best knowledge, no study investigating the synthesis or secretion of this mutant has been published as of now.

### Existence of novel biologically inactive human leptin mutants veiled by unsuspicious serum leptin levels

As presented in the preceding paragraphs, the serum leptin levels reported in the patients carrying homozygous leptin mutations have been close to undetectable or very low and thus out of proportion to the body fat mass found in these patients, making these patients severely leptin deficient [13,15,18,20-23].

A comparable situation is observed in leptin-deficient *obese* (*ob*/*ob*) mouse strains, in which leptin was originally discovered in 1994 [10,29]. In the *ob*^1J^ strain, a transition of the cytosine at position 12850 of the leptin gene to thymine (g.12850C>T) corresponding to position 313 of the transcript (c.313C>T) leads to a nonsense mutation of the protein, converting the arginine at position 105 to a stop (p.R105*) [10] [Ensembl:ENSMUSG00000059201, Ensembl:ENSMUST00000069789]. In this strain, the abundance of the leptin transcript in adipose tissue is drastically increased, while no corresponding leptin protein can be found in the serum, arguing for the synthesis of a mutant protein affected by an impaired intracellular transport and secretion [10,30]. In contrast, in the *ob*^2J^ strain, the insertion of a retroviral-like transposon featuring several splice acceptor and polyadenylation sites into the first intron of the leptin gene results in the generation of chimeric transcripts and consequently no leptin protein synthesis at all [10,29].

Most strikingly, by far, not all known murine leptin mutants exhibit a defective leptin synthesis and/or secretion.

*N*-ethyl-*N*-nitrosourea (ENU)-driven mutagenesis led to the generation of a leptin-mutant mouse strain displaying a coexistence of high serum leptin levels and a phenotype very similar to that of the *obese* mouse strains [30]. In this strain, a missense mutation of the leptin protein changing the valine at position 145 to a glutamate (p.V145E) was detected, which was caused by a transversion of the thymine at position 12971 of the leptin gene to an adenine (g.12971T>A) corresponding to position 434 of the transcript (c.434T>A) [30] [Ensembl:ENSMUSG00000059201, Ensembl:ENSMUST00000069789].

In two extensive *in vitro* studies, Peelman et al. and Iserentant et al. generated a large number of distinct murine leptin mutants featuring either single or multiple amino acid changes, essentially covering the whole surface of the leptin protein [31,32]. Interestingly, using COS-1 cells as a eukaryotic expression system, all mutants except for those targeting p.L34 were found to be synthesized and secreted to the cell supernatant, from which they could be purified for further experiments [31,32]. Several of these mutants showed pronounced changes in their capacity to bind and/or activate the leptin receptor, essentially proving that mutations rendering murine leptin biologically inactive do not necessarily have to affect leptin synthesis and/or secretion [31,32].

This raises the question whether forms of human congenital leptin deficiency exist, in which the production of biologically inactive leptin is veiled by the presence of unsuspicious or even elevated serum leptin levels.

Referring to current clinical recommendations, a sequencing of the leptin gene in patients with extreme early-onset obesity is suggested only in the presence of undetectable or very low serum leptin levels [6]. Therefore, it cannot be excluded that cases of congenital leptin deficiency with clearly detectable levels but bioinactive hormone exist in humans.

Diseases caused by bioinactive hormones are very rare, but still existent. For example, cases of biologically inactive protein have been reported for hormones like ACTH, TSH, as well as the leptin-related GH [33-36]. Therefore, the existence of congenital leptin dysfunction should definitely be considered.

## Conclusions

Congenital leptin deficiency caused by homozygous mutations in the leptin gene results in impaired satiety, intense hyperphagia, and extreme early-onset obesity accompanied by multiple metabolic, hormonal, and immunological abnormalities. In humans, eight distinct leptin mutations characterized by undetectable to low serum leptin levels have been identified so far. Mechanistically, defects in the synthesis and/or secretion of the hormone have been proposed and demonstrated for some of these mutations. Affected patients can be successfully treated with recombinant human leptin. Current clinical recommendations suggest sequencing of the leptin gene in cases of extreme early childhood obesity preferably in the presence of undetectable to low serum leptin levels. Thus, a change in practice might be necessary to identify novel leptin mutations not showing defects in the synthesis and secretion but in the biological activity of the hormone.

## Abbreviations

ACTH: adrenocorticotropic hormone
aa: amino acid
bp: base pair
CHO: Chinese hamster ovary cell line
COS-1, COS-7: CV-1 origin SV40 cell lines 1 and 7
ELISA: enzyme-linked immunosorbent assay
ENU: *N*-ethyl-*N*-nitrosourea
G-CSF: granulocyte colony-stimulating factor
GH: growth hormone
HEK293: human embryonic kidney 293 cell line
HGVS: Human Genome Variation Society
IL-6: interleukin 6
LBD: leptin-binding domain
*LEP*, *OB*: human leptin (obese) gene
NMD: nonsense-mediated mRNA decay
*ob*/*ob*: *obese* mouse strain
*ob*^1J^, *ob*^2J^: *obese* mouse strain variants
ORF: open reading frame
RIA: radioimmunoassay
TSH: thyroid-stimulating hormone
UTR: untranslated region

## References

[1] Wabitsch M, Moss A, Kromeyer-Hauschild K (2014). Unexpected plateauing of childhood obesity rates in developed countries. BMC Med.

[2] Ramachandrappa S, Farooqi IS (2011). Genetic approaches to understanding human obesity. J Clin Invest.

[3] Mantzoros CS, Magkos F, Brinkoetter M, Sienkiewicz E, Dardeno TA, Kim SY, Hamnvik OP, Koniaris A (2011). Leptin in human physiology and pathophysiology. Am J Physiol Endocrinol Metab.

[4] Paz-Filho G, Wong ML, Licinio J (2011). Ten years of leptin replacement therapy. Obes Rev.

[5] Farooqi IS, Matarese G, Lord GM, Keogh JM, Lawrence E, Agwu C, Sanna V, Jebb SA, Perna F, Fontana S, Lechler RI, DePaoli AM, O'Rahilly S (2002). Beneficial effects of leptin on obesity, T cell hyporesponsiveness, and neuroendocrine/metabolic dysfunction of human congenital leptin deficiency. J Clin Invest.

[6] Farooqi IS (2006). The severely obese patient--a genetic work-up. Nat Clin Pract Endocrinol Metabol.

[7] Dubern B, Clement K (2012). Leptin and leptin receptor-related monogenic obesity. Biochimie.

[8] von Schnurbein J, Heni M, Moss A, Nagel SA, Machann J, Muehleder H, Debatin KM, Farooqi S, Wabitsch M (2013). Rapid improvement of hepatic steatosis after initiation of leptin substitution in a leptin-deficient girl. Horm Res Paediatr.

[9] von Schnurbein J, Moss A, Nagel SA, Muehleder H, Debatin KM, Farooqi IS, Wabitsch M (2012). Leptin substitution results in the induction of menstrual cycles in an adolescent with leptin deficiency and hypogonadotropic hypogonadism. Horm Res Paediatr.

[10] Zhang Y, Proenca R, Maffei M, Barone M, Leopold L, Friedman JM (1994). Positional cloning of the mouse obese gene and its human homologue. Nature.

[11] Gong DW, Bi S, Pratley RE, Weintraub BD (1996). Genomic structure and promoter analysis of the human obese gene. J Biol Chem.

[12] Zhang F, Basinski MB, Beals JM, Briggs SL, Churgay LM, Clawson DK, DiMarchi RD, Furman TC, Hale JE, Hsiung HM, Schoner BE, Smith DP, Zhang XY, Wery JP, Schevitz RW (1997). Crystal structure of the obese protein leptin-E100. Nature.

[13] Montague CT, Farooqi IS, Whitehead JP, Soos MA, Rau H, Wareham NJ, Sewter CP, Digby JE, Mohammed SN, Hurst JA, Cheetham CH, Earley AR, Barnett AH, Prins JB, O'Rahilly S (1997). Congenital leptin deficiency is associated with severe early-onset obesity in humans. Nature.

[14] Rau H, Reaves BJ, O'Rahilly S, Whitehead JP (1999). Truncated human leptin (delta133) associated with extreme obesity undergoes proteasomal degradation after defective intracellular transport. Endocrinology.

[15] Strobel A, Issad T, Camoin L, Ozata M, Strosberg AD (1998). A leptin missense mutation associated with hypogonadism and morbid obesity. Nat Gene.

[16] Boute N, Zilberfarb V, Camoin L, Bonnafous S, Le Marchand-Brustel Y, Issad T (2004). The formation of an intrachain disulfide bond in the leptin protein is necessary for efficient leptin secretion. Biochimie.

[17] Chekhranova MK, Karpova SK, Yatsyshina SB, Pankov JA (2008). A new mutation c.422C>G (p.S141C) in homoand heterozygous forms of the human leptin gene. Russ J Bioorg Chem.

[18] Mazen I, El-Gammal M, Abdel-Hamid M, Amr K (2009). A novel homozygous missense mutation of the leptin gene (N103K) in an obese Egyptian patient. Mol Genet Metab.

[19] Niv-Spector L, Shpilman M, Grupi A, Gertler A (2010). The obese phenotype-inducing N82K mutation in human leptin disrupts receptor-binding and biological activity. Mol Genet Metab.

[20] Fischer-Posovszky P, von Schnurbein J, Moepps B, Lahr G, Strauss G, Barth TF, Kassubek J, Muhleder H, Moller P, Debatin KM, Gierschik P, Wabitsch M (2010). A new missense mutation in the leptin gene causes mild obesity and hypogonadism without affecting T cell responsiveness. J Clin Endocrinol Metab.

[21] Fatima W, Shahid A, Imran M, Manzoor J, Hasnain S, Rana S, Mahmood S (2011). Leptin deficiency and leptin gene mutations in obese children from Pakistan. Int J Pediatr Obes.

[22] Saeed S, Butt TA, Anwer M, Arslan M, Froguel P (2012). High prevalence of leptin and melanocortin-4 receptor gene mutations in children with severe obesity from Pakistani consanguineous families. Mol Genet Metab.

[23] Thakur S, Kumar A, Dubey S, Saxena R, Peters A, Singhal A (2013) A novel mutation of the leptin gene in an Indian patient. Clin Genet. doi:10.1111/cge.12289 10.1111/cge.12289 24304187

[24] Gibson WT, Farooqi IS, Moreau M, DePaoli AM, Lawrence E, O'Rahilly S, Trussell RA (2004). Congenital leptin deficiency due to homozygosity for the Delta133G mutation: report of another case and evaluation of response to four years of leptin therapy. J Clin Endocrinol Metab.

[25] Farooqi IS (2008). Monogenic human obesity. Front Horm Res.

[26] Saeed S, Bech PR, Hafeez T, Alam R, Falchi M, Ghatei MA, Bloom SR, Arslan M, Froguel P (2014). Changes in levels of peripheral hormones controlling appetite are inconsistent with hyperphagia in leptin-deficient subjects. Endocrine.

[27] Ozata M, Ozdemir IC, Licinio J (1999). Human leptin deficiency caused by a missense mutation: multiple endocrine defects, decreased sympathetic tone, and immune system dysfunction indicate new targets for leptin action, greater central than peripheral resistance to the effects of leptin, and spontaneous correction of leptin-mediated defects. J Clin Endocrinol Metab.

[28] Paz-Filho GJ, Babikian T, Asarnow R, Delibasi T, Esposito K, Erol HK, Wong ML, Licinio J (2008). Leptin replacement improves cognitive development. PloS one.

[29] Moon BC, Friedman JM (1997). The molecular basis of the obese mutation in ob2J mice. Genomics.

[30] Hong CJ, Tsai PJ, Cheng CY, Chou CK, Jheng HF, Chuang YC, Yang CN, Lin YT, Hsu CW, Cheng IH, Chen SY, Tsai SJ, Liou YJ, Tsai YS (2010). ENU mutagenesis identifies mice with morbid obesity and severe hyperinsulinemia caused by a novel mutation in leptin. PloS one.

[31] Peelman F, Van Beneden K, Zabeau L, Iserentant H, Ulrichts P, Defeau D, Verhee A, Catteeuw D, Elewaut D, Tavernier J (2004). Mapping of the leptin binding sites and design of a leptin antagonist. J Biol Chem.

[32] Iserentant H, Peelman F, Defeau D, Vandekerckhove J, Zabeau L, Tavernier J (2005). Mapping of the interface between leptin and the leptin receptor CRH2 domain. Journal of Cell Science.

[33] Medeiros-Neto G, Herodotou DT, Rajan S, Kommareddi S, de Lacerda L, Sandrini R, Boguszewski MC, Hollenberg AN, Radovick S, Wondisford FE (1996). A circulating, biologically inactive thyrotropin caused by a mutation in the beta subunit gene. J Clin Invest.

[34] Takahashi Y, Shirono H, Arisaka O, Takahashi K, Yagi T, Koga J, Kaji H, Okimura Y, Abe H, Tanaka T, Chihara K (1997). Biologically inactive growth hormone caused by an amino acid substitution. J Clin Invest.

[35] Samuels ME, Gallo-Payet N, Pinard S, Hasselmann C, Magne F, Patry L, Chouinard L, Schwartzentruber J, Rene P, Sawyer N, Bouvier M, Djemli A, Delvin E, Huot C, Eugene D, Deal CL, Van Vliet G, Majewski J, Deladoey J, Consortium FC (2013). Bioinactive ACTH causing glucocorticoid deficiency. J Clin Endocrinol Metab.

[36] Petkovic V, Miletta MC, Boot AM, Losekoot M, Fluck CE, Pandey AV, Eble A, Wit JM, Mullis PE (2013). Short stature in two siblings heterozygous for a novel bioinactive GH mutant (GH-P59S) suggesting that the mutant also affects secretion of the wild-type GH. Eur J Endocrinol.

